# Neurasthenia and Its Cure

**Published:** 1919-08-30

**Authors:** 


### NEURASTHENIA AND ITS CURE.

### The Effect of Handicraft on Mind and Body.

A paper of outstanding interest was read by Captain A. J. Brock, R.A.M.C.—"The Effect of Handicraft on Mind and Body"—to the Conference of New Ideals in Education at Cambridge on July 24. Treatment of shell-shocked men has led Captain Brock to look for some of the causes of increased neurasthenia, whether from war-shock or of gradual growth, in our educational systems. War conditions, he found, showed that many people are unfit to face unexpected circumstances, many know how to make a living, yet have never *learned to live*. We have assumed that all people need for happiness is satisfactory circumstances. People have therefore combined so as to secure these by legal enactments. Some men found themselves in the war. But trouble *makes or breaks*, and some after war experiences seem, as it were, "winded." The power of response seems gone; change, fresh air, exercise, fail to bring it back. A large number of young men in this state simply do not want to act. Hypnotism or suggestion may relieve insomnia, psychotherapy or stimulant keep the head temporarily upstream, but re-education is needed.

### Existence before the War.

This kind of neurasthenia is not due merely to shell-shock; it occurs in men living camp life and in civilians, and existed before the war. Now it is spreading; we may all be slightly tainted. The pre-disposition may be partly inherited, partly due to imperfect education. Thrown into the battle of war, or of life, a man may be "apostate," *vaincu de la vie*, his actions merely irrelevant. He may neglect his family, hardly write to them; to those round him, be self-centred, or merely a boon companion; may refuse to think of the past or the future. He may have lived through the terrors of the trenches and then the horror of a shell-burst, or may become nervous and dissipated in an army of occupation. He is not interested. Is he helped to be? At Paris and Mentone the speaker found libraries filled with light fiction to distract the men from the monotony of life, but not one book that pointed out the interest of their surroundings. In Paris men went to the same places of dissipation as they might in England; there was "nowhere else to go." For some of these education may now come too late. Similarly, with regard to work. War duties are not natural to life, but superimposed, and increase the tendency to view all work as an unpleasant necessity best turned over to subordinates. Then a man has no duties, domestic or social. Should he not prepare to take some up? All round him life is going on; he needs a way of entry into it.

### Essentials of Treatment.

As to treatment. Some symptoms of such cases are Nature letting off steam—as hysterical fits. Prescribed remedies may be of the bread-pill order. But re-education must follow, not merely of the mind, as may suit a healthy child; not preparing a young man for an examination, and sending him home with a certificate. He must learn to *live*, he has been merely existing, and that on dopes. True, some shocks will shatter the stoutest nerves, but these recover well; the trouble is with those who never got to grips with life, who are organisms detached from their surroundings. In a hospital near a great town, where geological conditions cause great variety of industries, he inquired into the past history of patients, their former work, intentions, ambitions, and tried to fit them into work that suited. Some at first were quite unable to fix their attention, a jig-saw puzzle served as a beginning. Many said they would begin "when better." His line of argument was, "This is the work you plan to do. Begin here and now. Put your own personality into it. Do not think about making money. There are two kinds of work. One "takes it out of you," the other "puts it in." You think you cannot make the effort? You mean you feel it difficult. When better you may be more selfish, have less energy." Work, again, must not be isolated, but related to the life of the district as a whole. If studied historically, industry is seen as a vital process, and from the past the mind looks to the future. Nor is regional study useless to one who will settle elsewhere. Man is a social animal, the neurasthenic grows unsociable. He encourages patients to have their families near, and to enter into the social life of the town, not merely its "purse-emptying" attractions. Thus life becomes something more than a clash of isolated atoms (a view which might be applied to States, showing Prussia an atom isolated and clashing)—pottery, painting, rug-making prove useful beginnings, it is sometimes difficult to tear a man away, and absorption in his own job becomes a danger. Artistic work awakens the neurasthenic to live, but a greater need is to learn the art of life. Happiness depends on the use an organism makes of its faculties. Here our education is at fault. Young people are not encouraged to open their eyes and look round.

### The Art of Life.

The art of life expresses the whole personality, which reacts to its whole environment. Doctors foster the tendency to isolation if they regard persons as cases rather than as human beings. Tommy will relate with pride that his disease is P.U.O., unaware that his pyrexia is merely of unknown origin, N.Y.D.—not yet diagnosed—impresses him quite as much. Politicians, perhaps, separate off their nations similarly, and modern concepts, such as capital and labour, man and woman, set people in opposition who have probably much the same ideals, such perhaps as "more money and less work!" Here is the first of the causes that lead up to war. Let the young study these causes; their spiritual director, the press, will not teach them. "Causes of tubercle" are learnt by studying many cases, they are found to be bad housing, etc. So with the study of many wars. In Paris they talk now of revolutions in Russia and Germany; in the light of their own history they may see the causes and find a prophylaxis against social disease. Such knowledge is vital education for life. It must be grasped that work is life and leads man to God, the Great Worker. It is not merely useful for keeping people out of mischief, but, on the other hand, if it becomes drudgery that is the fault of the worker. Teach young people to *use* their circumstances rather than hope only to change them, and to correlate their life with that of their neighbours and neighbourhood.

A speaker, in the ensuing discussion, remarked that man's deepest desire was supposed to be for love. He thought the deepest of all was the craving to be used by the community to which the man belongs.

